# A patient with Graves' disease who survived despite developing thyroid storm and lactic acidosis

**DOI:** 10.3109/03009734.2010.486908

**Published:** 2010-10-27

**Authors:** Tetsuhiro Yoshino, Daisuke Kawano, Takeo Azuhata, Tsukasa Kuwana, Rikimaru Kogawa, Atsushi Sakurai, Katsuhisa Tanjoh, Tatsuo Yanagawa

**Affiliations:** ^1^Department of Internal Medicine, Nerima General Hospital, Asahigaoka, Nerima-ku, Tokyo,Japan; ^2^Division of Emergency and Critical Care Medicine, Department of Acute Medicine, Nihon University School of Medicine, Oyaguchi Kamimachi, Itabashi-ku, Tokyo,Japan

**Keywords:** Graves' disease, hypoglycemia, lactic acidosis, thyroid storm

## Abstract

A 56-year-old woman with Graves' disease presented with the complaints of diarrhea and palpitations. Physical examination and laboratory data revealed hypothermia and signs of mild hyperthyroidism, heart failure, hepatic dysfunction with jaundice, hypoglycemia, and lactic acidosis. The patient was diagnosed as having developed the complication of thyroid storm in the absence of marked elevation of the thyroid hormone levels, because of the potential hepatic and cardiac dysfunctions caused by heavy alcohol drinking. A year later, after successful treatment, the patient remains well without any clinical evidence of heart failure or hepatic dysfunction. Thyroid storm associated with lactic acidosis and hypothermia is a serious condition and has rarely been reported. Prompt treatment is essential even if the serum thyroid hormone levels are not markedly elevated. We present a report about this patient, as her life could eventually be saved.

## Introduction

Thyroid storm is a serious complication of thyrotoxicosis, with a high mortality, that is diagnosed based on clinical examination (1,2). The severity of thyroid storm is not necessarily correlated with the thyroid hormone levels, although the exact underlying mechanisms explaining the severity are unknown (1,2). We encountered a patient who developed thyroid storm associated with lactic acidosis and hypothermia in the absence of marked elevation of the serum thyroid hormone levels. Thyroid storm associated with lactic acidosis and hypothermia is a serious condition and has rarely been reported. The case is discussed with references to the literature.

## Case report

The patient was a 56-year-old woman, with a personal history of habitually drinking 100 g or more of alcohol daily from 20 to 53 years of age and smoking 0.5 pack a day for 36 years. Past and family history was not contributory.

### Present illness

A 56-year-old woman was diagnosed as having Graves' disease (GD) at the age of 49, and was started on treatment with methimazole (MMI). After the thyroid hormone levels returned to the normal range, the patient discontinued her visits to the hospital. When she was 53 years old, she visited our hospital again with tachycardia and abdominal distension. Laboratory examination revealed evidence of mild hyperthyroidism (free thyroxine (fT4), 2.46 ng/dL) and hepatic dysfunction (total bilirubin (T.Bil), 0.5 mg/dL; aspartate aminotransferase (AST), 582 IU/L; and alanine aminotransferase (ALT), 323 IU/L). Abdominal Computed Tomography (CT) revealed ascites; however, there were no clear signs of liver cirrhosis. Based on the diffuse wall motion abnormality in the heart observed on echocardiography, the patient was diagnosed as having dilated cardiomyopathy (ejection fraction (EF), 38%). In addition to hyperthyroidism, alcohol was considered as one of the causes of the cardiomyopathy, in view of the history of heavy alcohol drinking. Treatment with iodine potassium (KI), MMI, propranolol (30 mg daily) and furosemide was initiated. The patient showed clinical improvement within a week; however, she discontinued all treatment after being discharged from the hospital.

In July 2008, she visited our hospital again with the complaints of palpitations and abdominal distension. Based on the clinical and laboratory data (serum free tri-iodothyronine (fT3), 12.7 pg/dL; fT4, 5.09 ng/dL), the patient was diagnosed as having developed recurrence of GD and congestive heart failure. Treatment was initiated on an outpatient basis with MMI (15 mg), furosemide, and spironolactone. One month later, the serum fT4 decreased to 0.4 ng/dL, suggestive of hypothyroidism. MMI was discontinued, and 2 weeks after the drug withdrawal the patient visited our hospital with the complaints of diarrhea and palpitations. Clinical and laboratory examination revealed evidence of mild hyperthyroidism again and of hepatic dysfunction with jaundice. Hepatic congestion secondary to hyperthyroidism and heart failure was diagnosed, and treatment with MMI (20 mg/day), metoprolol (20 mg/day), spironolactone, and furosemide was initiated. However, since the patient had persistent dyspnea for several hours after the start of the treatment, she was examined at the department of emergency medicine of our hospital and hospitalized.

### Condition at the time of admission

Glasgow coma scale score for consciousness, 15 (E4V5M6); blood pressure, 107/80 mmHg; pulse, 134 beats/min; body temperature, 34.5°C; respiratory rate, 30/min; oxygen saturation on room air, 95%. Physical examination revealed abdominal distention caused by ascites; however, there were no other abnormal findings, including edema of the legs.

### Chest X-ray

Cardiothoracic ratio, 61%; enlargement of the left ventricle.

### Electrocardiogram

Pulse rate, 134/min; atrial fibrillation +.

### Echocardiography

Left atrial dimension, 42 mm; mitral regurgitation, moderate; EF, 48%; left ventricular dimension–diastole, 41 mm; left ventricular dimension–systole, 31 mm; tricuspid regurgitation, trivial; estimated pulmonary arterial pressure, 40 mmHg; right atrial diameter, 44 mm; right ventricular diameter, 37 mm; inferior vena cava diameter, 23 mm; respiratory fluctuations, absent.

### Blood examination findings on admission (Table I)

T.Bil, 2.5 mg/dL; AST, 347 IU/L; ALT, 278 U/L, suggestive of hepatopathy with jaundice. Serum fT3, 7.80 pg/dL and serum fT4, 3.05 ng/dL, suggestive of hyperthyroidism. Serum N-terminal pro-B-type natriuretic peptide (NT-pro-BNP), 971 pg/mL, suggestive of heart failure. Arterial blood gas analysis (in ambient air) revealed a pH of 7.404, PCO_2_ of 10.0 mmHg, PO_2_ of 137.5 mmHg, HCO_3_ of 5.0 mEq/L, and Base Excess (BE) of −14.5, indicative of metabolic acidosis with respiratory alkalosis.

**Table I.** Laboratory Fidings.

Variable	On admission	16 hours later	Reference range
White blood cells (/μL)	8,700	21,300	3,500–8,500
Hemoglobin (g/dL)	13.5	11.8	11.5–15.0
Platelets (/μL)	224,000	195,000	150,000–350,000
C-reactive protein (mg/dL)	1.1	1.3	0–0.35
Albumin (g/dL)	3.9	3.5	3.9–5.2
Total bilirubin (mg/dL)	2.5	4.2	0.4–1.3
Creatine phosphokinase (IU/L)	657	2,169	50–170
Lactate dehydrogenase (U/L)	562	4,325	120–220
Aspartate aminotransferase (U/L)	347	2,751	10–35
Alanine aminotransferase (U/L)	278	1,036	5–40
Alkaline phosphatase (U/L)	981	791	100–320
γ-Glutamyltransferase (IU/L)	92	72	5–40
Serum creatinine (mg/dL)	0.74	1.77	0.4–0.8
Urea nitrogen (mg/dL)	17	25	8–20
Plasma glucose (mg/dL)	142	42	70–110
Free tri-iodothyronine (pg/mL)	7.80	4.03	2.0–4.5
Free thyroxine (ng/dL)	3.05	2.68	0.7–1.8
Thyroid-stimulating hormone (μU/mL)	<0.05	<0.05	0.3–4.5
Thyrotropin receptor antibody (IU/L)	13.3		0–0.9
N-terminal pro-brain natriuretic peptide (pg/mL)	971		0–125
Sodium (mmol/L)	131	126	136–145
Potassium (mmol/L)	4.5	5.8	3.6–4.8
Chloride (mmol/L)	98	90	99–107
pH	7.404	7.020	7.35–7.45
PaCO_2_ (mmHg)	10.0	17.0	35–45
PaO_2_ (mmHg)	137.5	135.0	80–100
Bicarbonate (mmol/L)	5.0	4.5	20–26
Base excess (mmol/L)	−14.5	−25.4	−3–3

### Course after admission

With the diagnosis of hyperthyroidism complicated by heart failure and metabolic acidosis, treatment was initiated with KI, MMI, furosemide, and spironolactone, in an attempt to improve the cardiohemodynamics. One dose of metoprolol (20 mg) was administered orally prior to hospitalization and then discontinued. After hospitalization, the patient's condition deteriorated, and 16 hours later she developed evidence of severe acidemia, hypoglycemia (42 mg/dL), and hepatopathy with jaundice (Table I). Signs of renal failure were also present, and the patient became delirious. On the basis of the Burch-Wartofsky score (2), which is a widely used global scale for evaluation of the severity of thyrotoxicosis, the patient's condition was diagnosed as thyroid storm (thyrotoxicosis with delirium (20 points), jaundice (20 points), moderate heart failure (10 points), and tachycardia (20 points); a total score of 61 or more is suggestive of definite thyroid storm). The patient was transferred to the Department of Emergency and Critical Care Medicine, Nihon University. At the time of admission to this department, the serum lactate level was 27.6 mmol/L, suggestive of lactic acidosis. Treatment with sodium bicarbonate, vitamin B1, dopamine, monoammonium glycyrrhizinate, L-arginine L-glutamate, lactulose, fresh frozen plasma, human atrial natriuretic peptide, and furosemide was started, and hemodialysis was instituted. The vital signs stabilized, and thereafter the patient, who was under treatment with oral MMI, furosemide, and ursodeoxycholic acid, was transferred to our hospital again. When the thyroid function parameters normalized, she was discharged from our hospital. Echocardiography repeated while the patient was in stable condition revealed an EF of 61%, and the serum brain natriuretic peptide was approximately 20 pg/mL. Serum biochemistry examination did not reveal any abnormalities. Currently, approximately a year after discharge from the hospital, the patient is leading a normal life without any subjective symptoms.

## Discussion

Thyroid storm is a serious complication of thyrotoxicosis, with a high mortality, that is diagnosed based on clinical examination (1,2). Our patient was diagnosed as having thyroid storm on the basis of the Burch-Wartofsky score (2), which is a widely used global scale for evaluation of the severity of thyrotoxicosis. The salient findings in our patient were as follows: deterioration of the clinical condition without marked elevation of the serum levels of thyroid hormones, lactic acidosis, and hypothermia. There are hardly any reports of thyroid storm associated with lactic acidosis in the literature (3–5). Hypothermia is also uncommon in thyroid storm; however, it has been reported sometimes in the elderly (2).

The severity of thyroid storm is not necessarily correlated with the thyroid hormone levels (1,2). Some factors such as sensitivity to catecholamines and advanced age have been implicated in the occurrence of thyroid storm, although the exact underlying mechanisms are unknown (1,6). Our present patient also developed thyroid storm without marked elevation of the serum fT4 levels (3.05 ng/dL).

Two possible etiological factors may be considered in our patient. The first is potential depression of cardiac function due to cardiomyopathy. When she developed combined left and right heart failure because of hyperthyroidism 3 years earlier, echocardiography had revealed evidence of cardiomyopathy, with an EF of 38%. When the thyroid function returned to normal, the EF increased to at least 60%, showing improvement of cardiac function; the serum brain natriuretic peptide also returned to within the normal reference range; there were no symptoms interfering with the activities of daily living. Both alcohol (7) and hyperthyroidism were considered to be etiologically related to the development of cardiomyopathy. Hyperthyroidism may lead to persistent or reversible cardiomyopathy; however, the precise underlying mechanism is unknown (8,9). In our patient, the cardiac function improved with improvement of the thyroid function, which could be explained as follows: the cardiac function may have been impaired by the alcoholic cardiomyopathy, and low cardiac output heart failure may have developed because of hyperthyroidism. The beta-blocker (metoprolol, 20 mg) administered for the treatment of tachycardia may have exacerbated the morbid condition.

The second factor that could be implicated in the development of thyroid storm in our patient, which resulted in worsening of the morbid condition, was alcoholic hepatopathy. Neither the serum albumin level and platelet count, nor abdominal CT revealed any definitive evidence of liver cirrhosis, but it is considered that the patient may have had hepatopathy, because she gave a history of heavy drinking of more than 100 g of alcohol every day for more than 30 years. In the presence of hyperthyroidism, pulmonary hypertension and right heart failure frequently lead to liver congestion (10), but it is considered that the condition progressed to the point of manifesting as jaundice because of the possible presence of alcoholic hepatopathy.

In regard to the etiology of thyroid storm, it is necessary to take into consideration the involvement of potential visceral disorders, including of the heart and liver, as in the present patient, as well as the thyroid hormone levels. Chronic heart failure with subjective symptoms, uncompensated liver cirrhosis, etc. associated with hyperthyroidism may easily lead to organ failure. It remains under debate whether such a condition should be diagnosed as thyroid storm. However, besides patients with organ dysfunction caused by alcohol, several elderly, diabetic patients and hypertensive patients also have decreased cerebral blood flow and myocardial disorders (11,12). When thyrotoxicosis precipitates failure of the hemodynamics in such patients, the possibility of development of thyroid storm is increased.

Our present patient showed lactic acidosis. Vitamin B1 was administered, because deficiency of this vitamin is one of the risk factors for the development of lactic acidosis. Vitamin B1 is a co-factor of decarboxylase in the tricarboxylic acid cycle, and its deficiency shifts glucose metabolism from aerobic towards anaerobic. With regard to acidosis complicating thyroid storm, while many reports have shown that thyroid storm can be complicated by ketoacidosis via the insulin antagonist actions of thyroid hormones (13), association of lactic acidosis with thyroid storm has rarely been reported (3–5). In our patient, all microbiological cultures were negative, and the condition improved without the administration of antibiotics. Therefore, the involvement of serious infectious disease in the etiology of our patient's condition was ruled out. Morbid conditions leading to lactic acidosis may include an increase in the rate of anaerobic metabolism caused by a decrease in the oxygen supply to the periphery as a result of decreased cardiac output and decrease in lactic acid metabolism caused by congestive hepatic failure; lactic acid is not converted into glucose due to dysfunction of the Cori cycle, resulting in worsening of lactic acidosis and development of hypoglycemia.

Patients with thyroid storm usually show elevation of the body temperature, but our present patient was hypothermic (body temperature, 34.5°C). Thyroid hormones act on the hepatic mitochondria to enhance utilization of the proton gradient for thermogenesis rather than for adenosine triphosphate (ATP) generation, which results in hyperthermia (1,14). The presence of hypothermia rather than hyperthermia in the present patient suggests that there was no proton gradient available for thermogenesis, because of the circulatory failure. A previously reported patient of thyroid storm with lactic acidosis was afebrile (4,5), and such an association is considered to be very serious. Hypothermia in a patient with thyroid storm indicates a very serious general condition indeed.

The data from the present patient suggest that even patients with mild hyperthyroidism may develop thyroid storm in the presence of severe depression of organ functions. Prompt treatment of patients with hypothermia and lactic acidosis is essential, because these conditions are suggestive of severe thyroid storm. Our present patient showed exacerbation of circulatory failure and lactic acidosis in the course of GD but eventually survived. We therefore considered the case as worthy of being reported.
